# The Effect of α-Arbutin on UVB-Induced Damage and Its Underlying Mechanism

**DOI:** 10.3390/molecules29091921

**Published:** 2024-04-23

**Authors:** Peng Shu, Yuan Wang, Lanyue Zhang

**Affiliations:** 1 HBN Research Institute and Biological Laboratory, Shenzhen Hujia Technology Co., Ltd., Shenzhen 518000, China; shupeng20@mails.ucas.ac.cn (P.S.); wangyuan@honeymate.cn (Y.W.); 2 State Key Laboratory Basis of Xinjiang Indigenous Medicinal Plants Resource Utilization, CAS Key Laboratory of Chemistry of Plant Resources in Arid Regions, Xinjiang Technical Institute of Physics and Chemistry, Chinese Academy of Sciences, Shihezi 830011, China; 3 University of Chinese Academy of Sciences, No. 19 (A) Yuquan Road, Shijingshan District, Beijing 100049, China; 4 School of Biomedical and Pharmaceutical Sciences, Guangdong Provincial Key Laboratory of Plant Resources Biorefinery, Guangdong University of Technology, Guangzhou 510006, China

**Keywords:** photoaging UVB radiation, skin whitening, α-arbutin, repair effect

## Abstract

Ultraviolet radiation can heighten tyrosinase activity, stimulate melanocyte production, impede the metabolism of numerous melanocytes, and result in the accumulation of plaques on the skin surface. α-Arbutin, a bioactive substance extracted from the arbutin plant, has been widely used for skin whitening. In this study, the whitening effect of α-arbutin by inhibiting tyrosinase activity and alleviating the photoaging effect induced by UVB are investigated. The results indicate that α-arbutin can inhibit skin inflammation, and its effectiveness is positively correlated with concentration. Moreover, α-arbutin can reduce the skin epidermal thickness, decrease the number of inflammatory cells, and down-regulate the expression levels of IL-1β, IL-6 and TNF-α, which are inflammatory factors. It also promotes the expression of COL-1 collagen, thus playing an important role in anti-inflammatory action. Network pharmacology, metabolomics and transcriptomics further confirm that α-arbutin is related to the L-tyrosine metabolic pathway and may interfere with various signaling pathways related to melanin and other photoaging by regulating metabolic changes. Therefore, α-arbutin has a potential inhibitory effect on UVB-induced photoaging and possesses a whitening effect as a cosmetic compound.

## 1. Introduction

The human skin, serving as the initial shield against environmental factors, has evolved a comprehensive immune system to shield its internal organs and tissues from chemical, physical, or microbial damage (Georgetti et al., 2013; Balupillai et al., 2018) [1,2]. The aging of skin is a multifaceted biological phenomenon, shaped by a combination of genetic and environmental elements. Such elements result in a gradual accumulation of physiological and structural alterations in the skin’s appearance. Ultraviolet radiation (UV) significantly accelerates the aging of the skin, with prolonged ultraviolet radiation-B (UVB) exposure frequently leading to the generation of reactive oxygen species (ROS) and harm to biomolecules like proteins and nucleic acids in the skin (Kammeyer and Luiten, 2015) [3]. The excessive exposure to UVB radiation also leads to the release of inflammatory cytokines such as Interleukin-6 (IL-6), Interleukin-1β (IL-1β), and Tumor Necrosis Factor-α (TNF-α) (Kim et al., 2012; Zhang, C et al., 2021; Jung et al., 2022) [4,5,6]. Melanocytes generate melanin as a protective response when subjected to UV rays. UV-induced damage is also involved in the formation of melanoma, and exposure to UVB accelerates skin aging and pigmentation (Baldea et al., 2009; Ma, L.-P et al., 2023) [7,8]. Additionally, these pro-inflammatory cytokines activate inflammatory cells to generate NADPH oxidase (Liu-Smith et al., 2015) [9], leading to the generation of ROS and oxidative stress in skin tissues. Consequently, this results in weakened immune systems, swelling, oxidative harm, aging, and a heightened likelihood of skin cancer. UVB radiation primarily induces premature skin aging and is considered a significant factor in the development of skin cancer. Recent studies have shown that several substances with anti-inflammatory biological activities possess protective effects against UVB-induced cell senescence (Choi E et al., 2019; Lu W et al., 2023) [10,11]. These findings suggest that inhibiting cellular processes or signaling pathways associated with UVB-induced cellular senescence may be a potential strategy for treating cellular senescence diseases resulting from excessive UVB exposure (Lim et al., 2023) [12].

Although there are a variety of sunscreen strategies available, including protective clothing, finding shade or staying indoors, applying sunscreen, etc., sunscreen is still the first choice (Koch, S. et al., 2017) [13]. At present, sunscreen clothing is usually evaluated by the transmittance of ultraviolet light through the fabric (Ultraviolet Protection Factor; UPF), and UPF > 50 is regarded as effective protection. There are two more common types of sunscreen: physical sunscreen and chemical sunscreen. Physical sunscreen is applied to the skin to form a physical sunscreen film, which directly reflects the UV rays, thus achieving the effect of sunscreen. Physical sunscreen is generally zinc oxide and titanium dioxide, which are generally not absorbed by the human body, so it is milder and safer to use, but its texture is thicker than chemical sunscreen, and it has obvious whitening after use and a slightly greasy and bad skin feeling. Chemical sunscreen is also known as an ultraviolet absorber, which is achieved by absorbing harmful ultraviolet rays and comprises ingredients such as benzophenone and ethyl hexyl salicylate. Because the molecules of chemical sunscreen are absorbed by the skin, the process of absorbing ultraviolet rays takes place inside the skin and is metabolized by the human body. At the same time, it will also cause some irritation to the skin (Dromgoole S H et al., 1990) [14]. Because of the huge heterogeneity of sunscreen products on the market in 2012, the European Commission determined several protection categories to classify the Sun Protection Factor (SPF) into ‘Low Protection’ (SPF 6, 10), ‘Medium Protection’ (SPF 15, 20, 25), ‘High Protection’ (SPF 30, 50) and ‘Very High Protection’ (SPF 50+). To label sunscreen, the product must provide at least 6 SPF (Sebastian Singer et al., 2019) [15]. It is worth noting that fps only represents the category of protection for UVB. In addition, PA is an acronym for Protection Grade of UVA, with “+” indicating the product’s ability to defend against long-wave ultraviolet rays. The PA grade is determined according to the long-wave ultraviolet protection index (Protection Factor of UVA, PFA) of sunscreen cosmetics, which reflects the protective effect of long-wave ultraviolet tanning and is a protective index to evaluate the ability of sunscreen cosmetics to prevent sunburn. The higher the PA level, the better the effect of preventing skin from tanning. China’s laws and regulations require the PA logo to be based on the actual PFA value of the product, and the range can be marked as PA+~PA++++ according to the sunscreen ability. Most sunscreens have good adsorption effects, but their anti-sweat and anti-sebum effects are poor. On the one hand, sweat directly washes off sunscreen, resulting in a decrease in film thickness; on the other hand, sunscreen is redistributed with the flow of sweat, resulting in reduced uniformity. Both of these mechanisms will have a negative impact on the activity of sunscreen and lead to a decrease in UV protection. The experimental studies show that after sweating for six hours, the SPF effect of sunscreen decreased from 50 to 30 (Keshavarzi F et al., 2021; Ruvolo E et al., 2020) [16,17].

Nowadays, there has been growing interest in plant-based ingredients as potential sunscreens due to their capacity to absorb UVB and strong antioxidant properties (Burns et al., 2013) [18]. Relative to “biological sunscreen,” “plant-derived sunscreen” research is more in-depth. The principle of “plant sunscreen” is similar to chemical sunscreen and physical sunscreen, mainly through flavonoids, anthraquinones and their derivatives, plant polyphenols and other components in plant extracts to absorb ultraviolet rays (Cooman, L et al., 1998) [19]. Therefore, natural medicines showing absorption spectra within the UVB range receive a lot of attention for their potential in preventing UVB-induced skin damage (Duan et al., 2019; Ng S Y et al., 2012; Saraf S et al., 2010) [20,21,22].

Arbutin is a hydroquinone glycoside (497-76-7). Because of its strong inhibitory effect on human tyrosinase activity, arbutin is used as a powerful whitening agent in the cosmetic industry. It is a natural compound found in many plants in the form of the isomerization of glycoside bonds between glucose and hydroquinone. α-arbutin (84380-01-8) is an isomer of natural arbutin. α-arbutin is usually produced by transglucylation of hydroquinone by microbial glycosyltransferase (Zhu X et al., 2018) [23]. Studies have indicated that arbutin can prevent tyrosinase activity, thereby preventing melanin production and reducing skin pigmentation (Won et al., 2012; Chunhakant and Chaicharoenpong, 2019; Ye et al., 2019; Zhang et al., 2022) [24,25,26,27]. Recent studies have shown that α-Arbutin shows potential anti-photoaging effects, likely linked to diminished cellular inflammation from IL-6 and TNF-α (Hazman Ö et al., 2021; Lee et al., 2012) [28,29]. The increase in MMP expression is one of the main effects of ultraviolet radiation on the skin. MMP is responsible for the degradation of ECM proteins such as collagen, fibronectin, elastin and proteoglycan. They also play an important role in tissue remodeling and repair. The excessive degradation of these proteins caused by excessive production of MMP-1, MMP-3, and MMP-9 will lead to photoaging of the skin, resulting in thick wrinkles and skin relaxation through the oxidation, decomposition, or destruction of collagen and elastin (Krystyna J et al., 2021) [30]. However, due to the complex and multi-target actions of the active components of natural plants, the pharmacological mechanism cannot be fully revealed by conventional means. Thus, exploring a plant’s manifold potential mechanisms warrants more efficient methods. 

In this study, we aimed to investigate whether α-arbutin can protect mice from UVB-induced UV damage. KM mice were irradiated with UVB, and the mice in different groups were smeared with corresponding drugs before irradiation. The effect of α-arbutin on UVB damage was studied by (Hematoxylin and eosin) HE staining, (Toluidine Blue) TB staining, MASSON staining, biochemical tests, Network pharmacology and metabonomics. The results show that the intrinsic protective mechanism of α-arbutin against UVB-mediated UV injury may be regulated by the inflammatory response, immune regulation pathway and downstream factors such as COL-I, IL-1 β, TNF- α and IL-6. This study provides a new insight into the protective mechanism of α-arbutin against UV damage.

## 2. Results

### 2.1. MTT Cytotoxic Assay 

**Figure 1:**
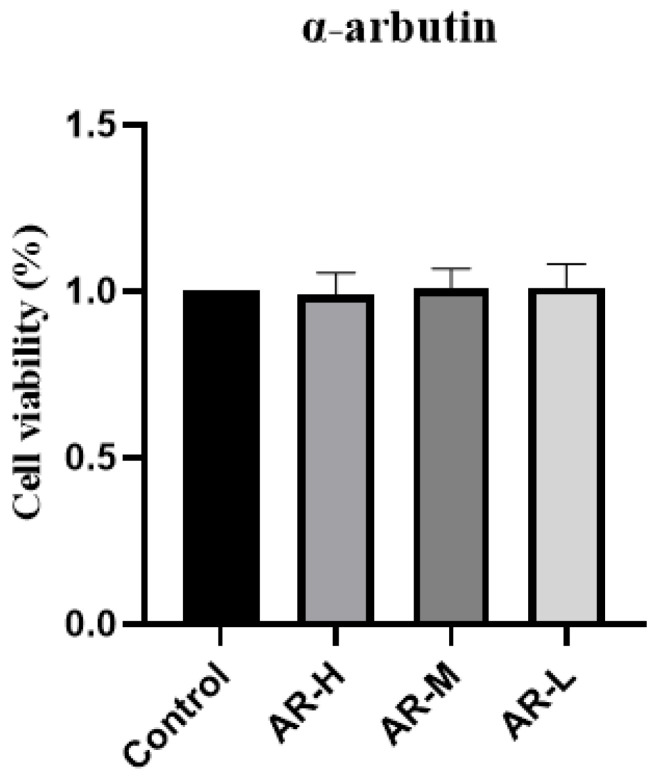
Survival rate of HaCaT cells in α-arbutin toxicity test.

The MTT (Methyl thiazolyl tetrazolium) method was used to determine the toxicity of three concentrations of α-arbutin to HaCaT cells. Extract concentrations that led to more than 90% of cell viability were considered safe. The results showed that α-arbutin with a gradient concentration of 0.5 mg mL was safe and non-toxic and could be used in follow-up experiments (Figure 1).

### 2.2. Animals and Treatment

**Figure 2:**
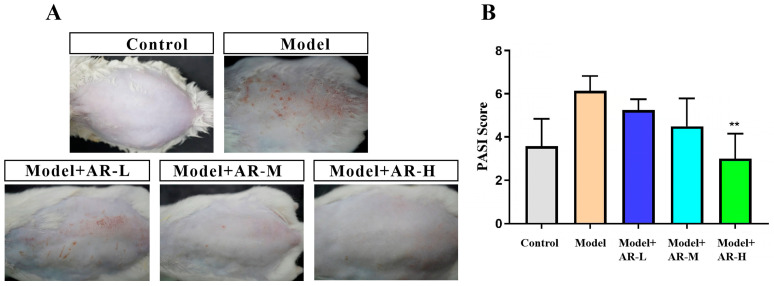
Image and analysis results of skin tissue on the back of mice. (**A**) Erythema and wrinkles on the back skin of mice after administration. (**B**) Statistical analysis results of visual scores on the back of mice. When evaluated by variance analysis and Duncan multiple range test, there were significant differences from the model (** *p* < 0.01). Each value represented the mean ± SD of 3 mice.

According to the evaluation standard of the back of mice (Cao Di et al., 2016) [31] and combined with the experiment (Table 1), we scored and summed the back skin of the control group, the model group, and the mice after administration on the two dimensions of erythema and wrinkles. Figure 2A depicts distinctions in murine back skin features (wrinkles and erythema) within five study groups: control group, model group, model + AR − L (arbutin-low), model + AR m (arbutin-medium), and model + AR − H (arbutin-high). Notably, ultraviolet (UV)-exposed mice exhibited elevated erythema and wrinkles; however, α-arbutin treatment considerably reduced these signs. Figure 2B, unveiled through comprehensive statistical analysis using GraphPad software (8.0.2) validated this significant finding. Specifically, a drastic increase in murine back erythema and wrinkle scores was observed for the model group compared to the control group. Equally noteworthy was the fact that the photoaging score of α-arbutin-treated mice was notably lower than that in the model group, indicating a recovery or protective impact against photoaging. Consequently, α-arbutin displayed remarkable potential as a therapeutic agent for murine photoaging.

### 2.3. Histological Analysis of HE Staining

**Figure 3:**
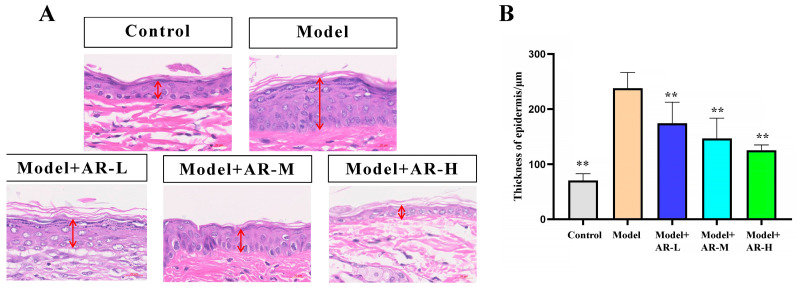
Histological evaluation of epidermal thickness of mouse back skin tissue. (**A**) Hematoxylin-eosin-stained mouse back epidermis. The red arrow represents the thickness of the epidermis. (**B**) Representative epidermal thickness for each treatment group. When evaluated by variance analysis and Duncan multiple range test, there were significant differences from the model (** *p* < 0.01). Each value represented the mean ± SD of 3 mice.

Epidermal hyperplasia, a hallmark of allergic reactions’ skin damage, serves as a gauge for assessing drug inhibitory potency against it. Image Pro Plus software (Version 6.0.0.260 for Windows 2000/XP Professional) was used to measure skin thickness. Measure the vertical distance between the shallowest part of the particle layer and the deepest part of the basal layer of the epidermis during measurement, and use μm as the unit of measurement. The HE slices are first scanned and archived using a slice scanner and then measured using the Image Pro Plus software’s built-in ranging function. As shown in Figure 3A, Compared with the control group, the skin thickness of the model group mice significantly increased, which is due to acute skin damage caused by UVB irradiation. Due to inflammatory reactions and edema, it is often manifested as an increase in skin thickness. In addition, it was observed that, compared to the model group, the model + AR − L group, the model + AR m group, and the model + AR − H group all exhibited a reduction in the epidermal thickness of mice’s skin. This indicates that the local application of the tested α-arbutin could effectively inhibit epidermal hyperplasia caused by skin allergies. This conclusion is also confirmed by Figure 3B.

### 2.4. Mast Cells Were Detected by Toluidine Blue

**Figure 4:**
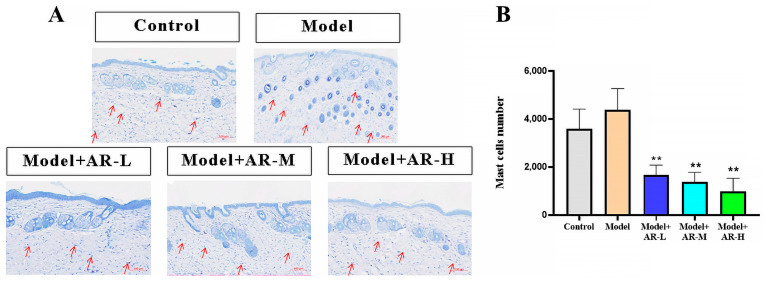
Evaluation of the number of mast cells in mouse back skin tissue. (**A**) Stained sections of mouse back skin tissue. The red arrow points to the location of mast cells. (**B**) The number of mast cells in mouse skin tissue was counted. After analysis of variance and Duncan multivariate range test, the difference was statistically significant compared with the model (** *p* < 0.01). Each value represents the mean ± SD of 3 mice.

As shown in Figure 4A, the blue-purple dots are colored mast cells. Figure 4B shows the low number of mast cells in the control group, with a dramatic increase post-UV exposure. These results indicate that UV can induce skin inflammation and increase mast cell numbers, and the number of mast cells in the group treated with α-arbutin significantly decreased (*p* < 0.01), proving that α-arbutin has an inhibitory effect on the development of UV-induced skin inflammation and a strong anti-photoaging effect.

### 2.5. Masson Staining

**Figure 5:**
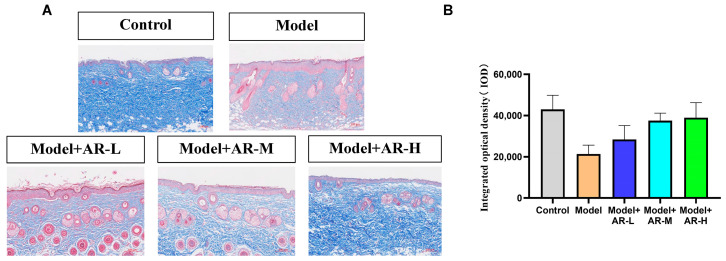
Evaluation of the number of collagen fibers in mouse back skin. (**A**) Masson staining of the dorsal epidermis of mice. Blue tissue represents collagen fibers and red tissue represents muscle fibers. (**B**) Epidermal thickness representative of each treatment group. After analysis of variance and Duncan multivariate range test, the difference was statistically significant compared with the model. Each value represents the mean ± SD of 3 mice.

Masson staining was utilized to assess the formation and deposition of collagen at different time intervals following UV irradiation skin injury models, which have been treated with various experimental sample groups. Each Masson-stained mouse skin specimen was randomly selected from three fields of view, and the integrated optical density (IOD) values of the blue-stained collagen fibers were calculated using Image-Pro Plus image analysis software (Version 6.0.0.260 for Windows 2000/XP Professional). The mean value was determined, and this semi-quantitative method was employed to represent the collagen fiber content. As depicted in Figure 5A, the skin tissue subjected to UV modeling exhibited reduced blue collagen tissue in the Masson staining as compared to the normal blank group. This phenomenon arises as UV possesses a potent penetrating ability, and during the modeling procedure, it infiltrates the skin surface and irradiates the dermis, impairing the skin’s free radicals and resulting in the breakage of collagen and elastin fibers in the tissue. Consequently, the proportion of blue collagen tissue in the UV modeling group was significantly inferior to that observed in the blank group. From Figure 5A, it can be observed that the collagen tissue in the model + AR − L, model + AR-M, and model + AR − H treatment groups exhibited varying degrees of increments, with model + AR − H demonstrating the most efficacious outcome as compared to the blue collagen tissue present in the model group. From Figure 5B, the c group exhibited superior reparative characteristics in the skin’s collagen tissue as compared to the other sample groups.

### 2.6. Immunohistochemistry

**Figure 6:**
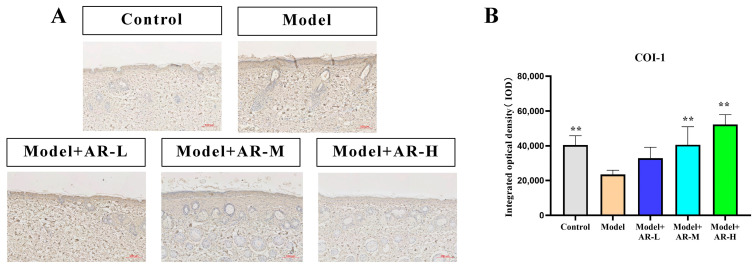
COL-1 immunohistochemical staining results of mouse back skin tissue. (**A**) COL-1 integrated optical density (IOD) values in different groups of mice. (**B**) N = mean ± SD of 3 mice per group, ** *p* < 0.01.

**Figure 7:**
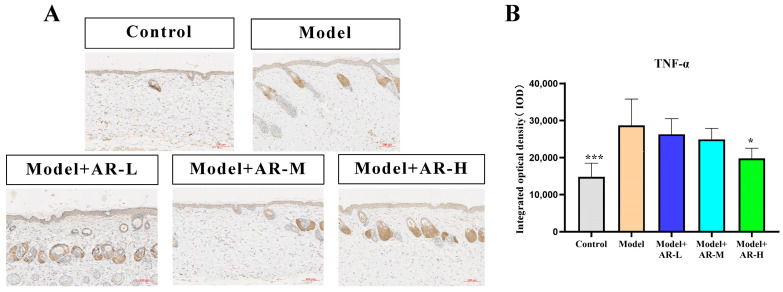
Immunohistochemical staining of TNF-α in mouse back skin. (**A**) TNF-α integrated optical density (IOD) values in different groups of mice. (**B**) N = mean ± SD of 3 mice per group, * *p* < 0.05 and *** *p* < 0.001.

**Figure 8:**
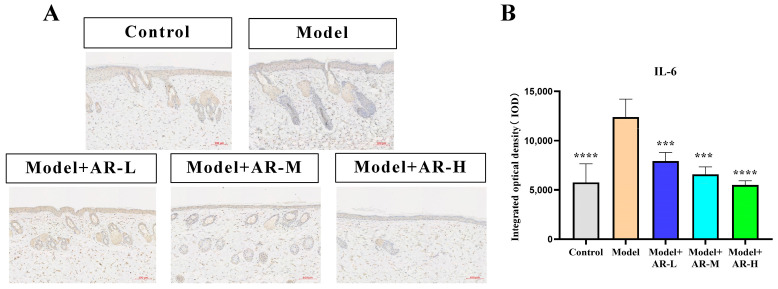
Immunohistochemical staining of IL-6 in mouse back skin. (**A**) IL-6 integrated optical density (IOD) values in different groups of mice. (**B**) N = mean ± SD of 3 mice per group, *** *p* < 0.001, **** *p* < 0.0001.

**Figure 9:**
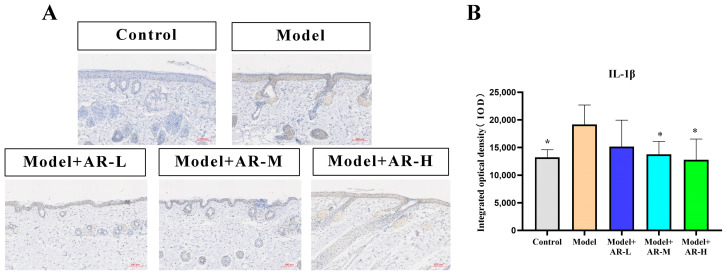
IL-1β immunohistochemical staining results of mouse back skin tissue. (**A**) IL-1β integrated optical density (IOD) values in different groups of mice. (**B**) N = mean ± SD of 3 mice per group, * *p* < 0.05.

TNF-α, IL-6, and IL-1β are cytokines associated with inflammation and play an important role in the production of skin inflammation. COL-1 is a collagen protein that is closely associated with skin aging. According to Figure 6, Figure 7, Figure 8 and Figure 9, the expression of TNF-α, IL-6, and IL-1β was significantly up-regulated in the model group. However, after treatment with three concentrations of α-arbutin, the expression of TNF-α, IL-6, and IL-1β decreased compared to the model group, while the expression of COL-1 increased (Figure 6). According to the results of the statistical calculation of the IOD of each group. The expression of TNF-α, IL-6, and IL-1β in mice treated with different concentrations of α-arbutin decreased significantly compared to the UV group, while the expression of COL-1 increased, which was statistically significant (*p* < 0.05). However, the expression levels of TNF-α, IL-6, and IL-1β in the different doses of the drug treatment groups were basically the same compared with the control group.

### 2.7. Metabolomics Analysis

**Figure 10:**
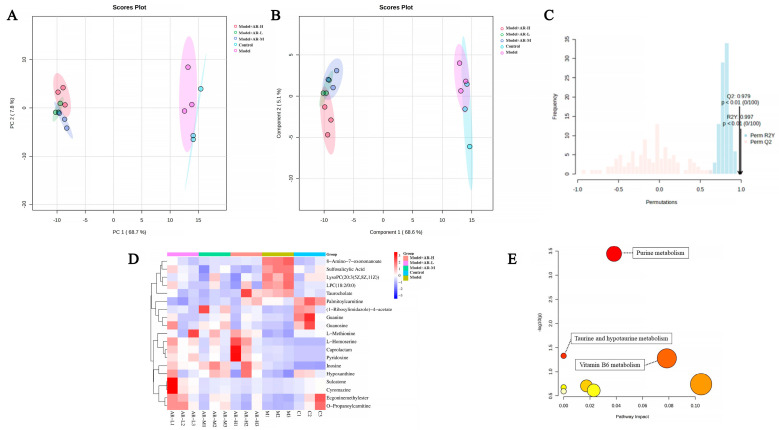
PCA (**A**) and PLS-DA (**B**) maps were used to distinguish differences in metabolic characteristics between different groups, respectively. (**C**) Replacement test diagram (R2Y represents the interpretation rate of the model in the *Y*-axis direction (or it can be understood as the square of the percentage of the original data information retained in the *X*-axis direction); Q2 represents the prediction rate of the model). (**D**) Heat maps. Red indicates high expression and blue indicates low expression. (**E**) Enrichment of metabolic pathways. The color depth of the points in the figure indicates the importance of metabolic pathways, with red indicating that metabolic pathways are more influential and light yellow indicating that metabolic pathways are less influential. The color of bubbles represents enrichment significance, the redder the color is, the stronger the significance is, the size of bubbles represents the number of genes enriched, and the larger the bubble is, the more genes are enriched.

To investigate the efficacy of α-arbutin in delaying skin aging, metabolite profiling across varying concentration levels was conducted using principal component analysis, followed by partial least squares discriminant analysis (PLS-DA) modeling to detect overall metabolic variance between the groups. As evident from Figure 10A,B, the control group and model group are obviously divided into two clusters, indicating that the model has been successfully established. Figure 10C confirms model validity via permutation testing post-1000 simulations with good predictive ability. Finally, 19 distinct differentially metabolited molecules (DMs) were discernible upon treatment, as shown in Figure 10D. Compared with the control group, four kinds of DMs, such as 8-amino-7-oxonanoate, sulfosalicylic acid, lysophosphatidylcholine (LPC) (18:2/0:0), and taurine, were down-regulated. In contrast, 15 kinds of DMs, such as L-methionine, L-homoserine, and caprolactam, were up-regulated. Interestingly, many of these DMs demonstrated significant alterations post-treatment, suggesting that these metabolites could regulate metabolic disorders to some extent. Following their analysis within Metabo Analyst, eight pathways were found to be enriched. The impacts were significant (*p* < 0.05), specifically in purine metabolism, taurine and low taurine metabolism, and vitamin B6 catabolism.

### 2.8. Transcriptome Analysis

#### 2.8.1. Analysis of Differentially Expressed Genes (DEGs)

**Figure 11:**
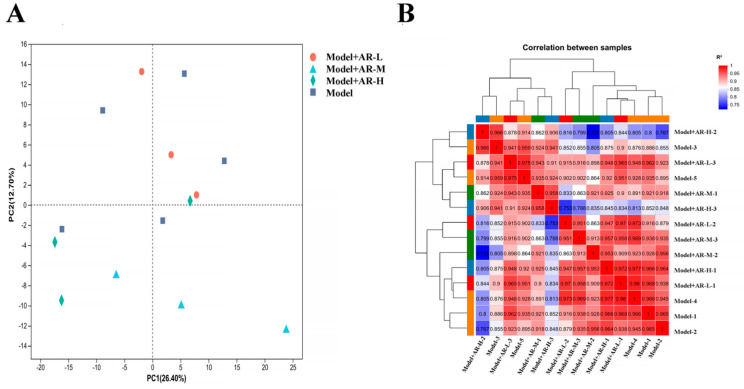
(**A**) Principal component analysis diagram; (**B**) Sample correlation.

In order to study the anti aging effect of α-arbutin on the skin, transcriptomic analysis was conducted on different concentration groups. The analysis of differentially expressed genes and levels of gene expression: Principal component analysis (PCA) is used to analyze the total genetic differences in a sample and the degree of similarity between samples within a group. The correlation of each group of samples is normal, and there are no changing samples in Figure 11A. In the correlation analysis of samples, the Pearson correlation coefficient (R2) was used as an evaluation index for biological replication. For each sample, as shown in Figure 11B, the closer the absolute value of R2 was to 1, the stronger the correlation between two replicated samples was.

**Figure 12:**
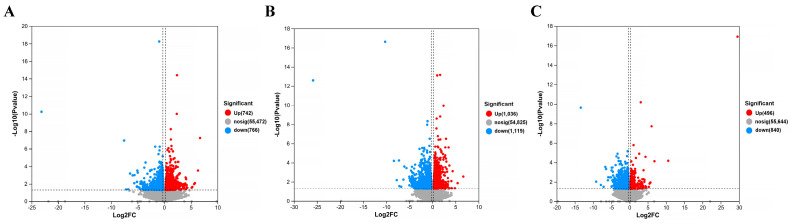
Volcanic plot of (**A**) model + AR − L vs. UV, (**B**) model + AR m vs. UV, and (**C**) model + AR − H vs. UV (*p* < 0.05).

**Figure 13:**
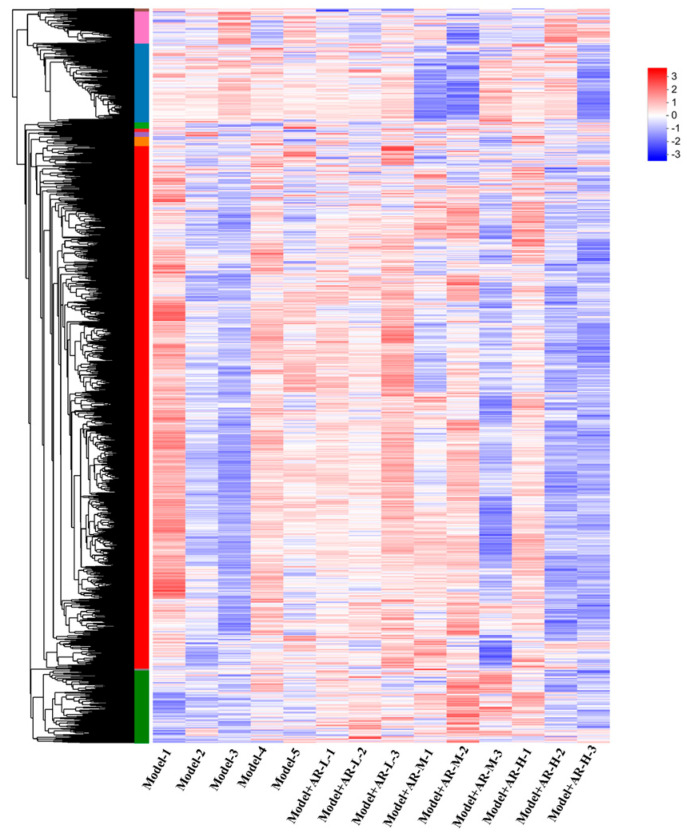
Gene expression heat maps of model + AR − L and UV, model + AR m and UV, and model + AR − H and UV (*p* < 0.05).

The gene screening threshold def was set at *p* < 0.05, |log2 FC| >1.2 for comparison. The differentially expressed genes (DEGs) between the treatment group and the model group were compared. The volcano map showed the total number of genes detected in the differential groups and the number of differential genes that were significantly up-regulated and down-regulated. The *X*-axis of the volcano map showed the change in gene expression multiples; the *Y*-axis showed the level of gene significance, with red dots representing up-regulated differential genes and blue dots representing down-regulated differential genes. Gray dots indicate non-differentially expressed genes. As can be seen from Figure 12, the model + AR m group had the most up-regulated gene difference multiples. The model + AR m group and the model + AR − H group had better gene significance, and most genes in the model + AR − L group were most obvious. Clustering heatmaps showed the differential genes and their clustering between samples. The horizontal coordinate represents sample information and the hierarchical clustering results, while the vertical coordinate represents the differential genes and their hierarchical clustering results. Red indicates high expression and blue indicates low expression. According to Figure 13, genes significantly expressed in the model group were significantly expressed in the treatment group, and this phenomenon was most obvious in the model + AR − L treatment group.

#### 2.8.2. Enrichment Analysis of GO Function and KEGG Pathway

**Figure 14:**
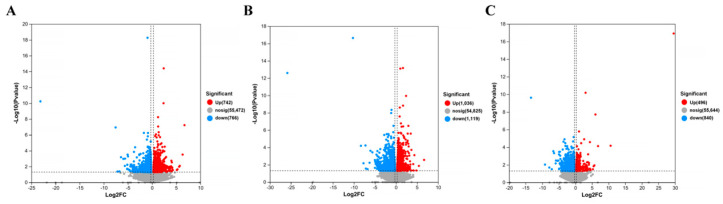
GO functions of (**A**) model + AR − L and UV groups, (**B**) model + AR m and UV groups, and (**C**) model + AR − H and UV groups.

**Figure 15:**
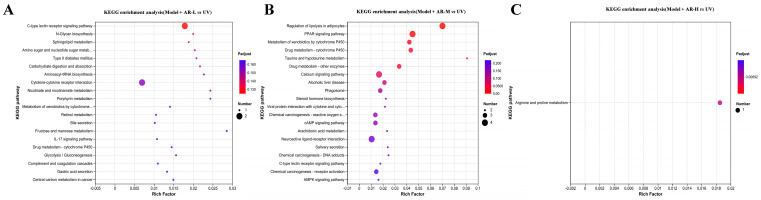
(**A**) KEGG enrichment in model + AR − L and UV groups, (**B**) model + AR m and UV groups, and (**C**) model + AR − H and UV groups.

Gene Ontology (GO) is an international standard classification system for gene function. GO is divided into three parts: molecular function, biological processes, and cell components, with the vertical coordinate being the GO secondary classification and the horizontal coordinate being gene expression. The Kyoto Encyclopedia of Genes and Genomes (KEGG) is a comprehensive database of genomes, biological pathways, diseases, drugs, chemicals, and other information. KEGG can be used to check in which pathways DEG is enriched and the up-down regulation relationship in this pathway. The ordinate represents the KEGG pathway, and the abscess represents the enrichment factor. The larger the enrichment factor, the greater the degree of enrichment. The larger the dot, the more pathways there are to enrich differential genes, and the redder the dot, the more significant the enrichment. As can be seen from Figure 14, GO gene enrichment was mainly manifested in the binding, cell part, and cellular process of the group model + AR − L, the cell part of the group model + AR-M, and the cell part of the group model + AR − H. These gene enrichment pathways are numerous and significantly enriched. KEGG enriched 20 signaling pathways in each control group, mainly related to inflammatory response, immune regulation, hormone synthesis and metabolism, vitamin regulation, and other signaling pathways. Specifically, the model + AR − L group was enriched with the UV group in terms of cytokine-cytokine receptor interactions. In Figure 15, the model + AR m and UV groups are enriched in chemical carcinogenic-receptor activation and neuroactive ligand-receptor interactions, while the model + AR − H and model-5 groups are closely related to arginine and proline metabolism.

### 2.9. Network Pharmacology Analysis

**Figure 16:**
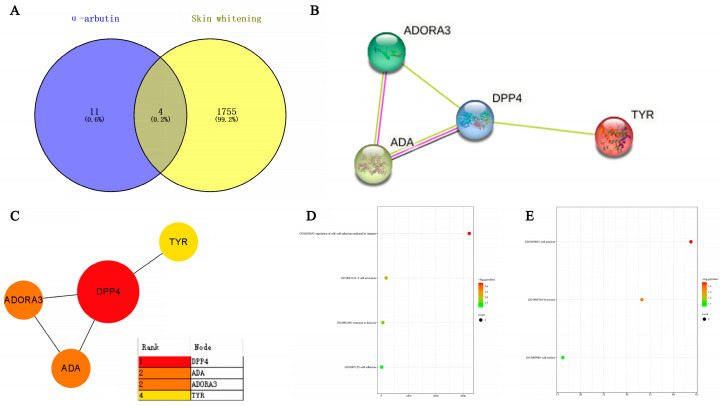
Network pharmacological analysis of α-arbutin on the whitening effect (**A**) Wayne diagram of α-arbutin and whitening intersection genes. (**B**,**C**) Diagram of the α-arbutin protein interaction network. Protein–protein interaction (PPI) represents the interaction between four common targets. (**D**,**E**) Bubble diagram of α-arbutin GO enrichment. The bubble on the left is BP and the bubble on the right is CC.

α-arbutin targets were predicted by Swiss Target Prediction. Using “Skin whitening and melanin” as keywords, the GeneCards database was searched to obtain whitening targets. There were 1755 results after weight removal. Then, the results were uploaded to the VENN diagram on an online website and submitted; a total of four intersection targets were obtained, and a Wayne (Venn) diagram was drawn at the same time. As shown in Figure 16A, the blue circle in the figure represents 11 targets that can be predicted by α-arbutin, while the yellow circle represents 1755 targets of skin aging. The intersection of the two circles represents the compounds potential targets for whitening, a total of four. After the shared protein genes were uploaded to the STRING website, a preliminary infographic of protein interactions was obtained, as shown in Figure 16B. Furthermore, the compound target network was constructed based on Cytoscape 3.7.2 software, and core nodes were selected according to network topological characteristics such as the node degree value, and the network diagram was drawn, as shown in Figure 16C. It was predicted that α-arbutin was correlated with DPP4 (Dipeptidyl Peptidase 4), TYR (Tyrosinase), ADA (Adenosine Deaminase), and ADORA3 (Adenosine A3 Receptor). DPP4 is a protein-coding gene involved in the synthesis, triage, inactivation, and protein metabolism of incretin. Additionally, DPP4 is also associated with cell surface glycoprotein receptors that mediate co-stimulatory signals necessary for T cell activation by the T cell receptor (TCR). TYR is a key target in the melanin production pathway, suggesting that α-arbutin is related to whitening. For each gene, its basic function is based on its protein domain and the literature that has been studied. GO is a database of gene-related functions based on different classification ideas. GO enrichment analysis was carried out with the help of the DAVID website (DAVID: Functional Annotation Tools (ncifcrf.gov)). A total of seven GO items were obtained, including four biological processes (BP) and three cell components (CC). For a more intuitive understanding of gene function, P values and Count were used as a reference for sorting, with the help of the microscopic generated enrichment bubble chart letter website (https://www.bioinformatics.com.cn/ (accessed on 10 April 2024)). Biological process analysis found that, as shown in Figure 16D, the key target genes are mainly involved in the regulation of cell–cell adhesion mediated by integrin, T cell activation, response to hypoxia, cell adhesion, etc. It can be seen from Figure 16E that the cell component analysis results suggest that the cell junction, lysosome, and cell surface are related.

**Figure 17:**
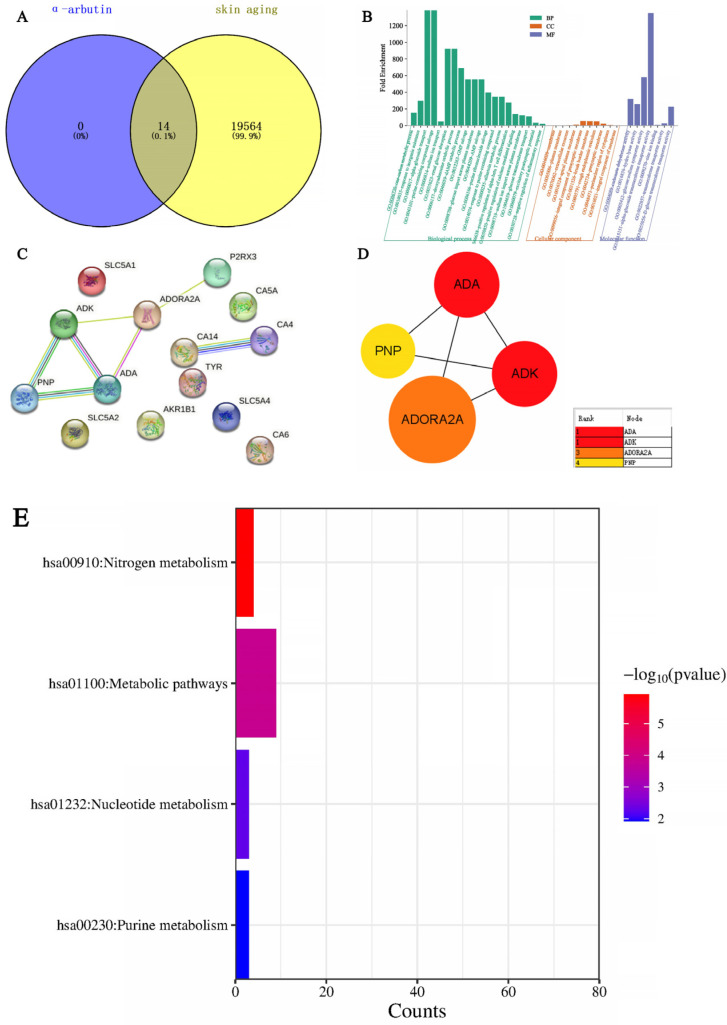
Network pharmacological analysis of the effect of α-arbutin on skin aging. (**A**) Wayne map of α-arbutin and whitening cross genes. (**B**) α-arbutin GO enrichment histogram. (**C**,**D**) α-arbutin protein interaction network diagram. PPI represents the interaction between four common goals. (**E**) KEGG bar chart.

Through SwissTarget Prediction (http://www.swisstargetprediction.ch/ (accessed on 11 April 2024)) to predict alpha-arbutin target 14, with “Skin aging” as keywords, GeneCards database search, get the target of skin aging. There were 19,564 results after weight removal. Then, the results were uploaded to the VENN Diagram online website and submitted; a total of 14 intersection targets were obtained, and the Venn diagram was drawn at the same time. As shown in Figure 17A, the blue circle in the figure represents 14 targets that can be predicted by alpha-arbutin. The yellow circle represents the 19,564 targets of skin aging. The intersection of the two circles represents a total of 14 potential targets of the compound for skin aging. GO enrichment analysis was carried out with the help of the DAVID website (DAVID: Functional Annotation Tools (ncifcrf.gov)), and 37 GO items were obtained, including 20 Biological Processes (BP), 10 Cell Components (CC), and 7 Molecular Functions (MF). For a more intuitive understanding of gene function, P values and Count were used as a reference for sorting, with the help of the microscopic letter website (https://www.bioinformatics.com.cn/ (accessed on 11 April 2024)) to generate a histogram. Biological process analysis found that, as shown in Figure 17B, key target genes are mainly involved in α-glucoside transport, purine-containing compound salvage, renal glucose absorption, deoxyadenosine catabolic process, glucose import across the plasma membrane, etc. The results of the cell component analysis showed that these targets were predominantly located on the membrane, plasma membrane, extracellular exosome, apical plasma membrane, integral component of the presynaptic membrane, brush border membrane, and rough endoplasmic reticulum. The results of molecular function analysis suggested that they were associated with carbonate dehydratase activity, hydrolase activity, glucose: sodium symporter activity, α-glucoside transmembrane transporter activity, zinc ion binding, transmembrane transporter activity, and D-glucose transmembrane transporter activity. After uploading the shared protein genes to the STRING website, a preliminary infographic of protein interactions was obtained, as shown in Figure 17C. Furthermore, the compound target network was constructed based on Cytoscape 3.7.2 software, and core nodes were selected according to network topological characteristics such as node degree value, and the network diagram was drawn, as shown in Figure 17D. α-arbutin was predicted to have a certain correlation with ADK, ADA, CA4, CA14, P2RX3, PNP, and ADORA2A. The core targets are ADA, PNP, ADK, and ADORA2A. ADK (Adenosine Kinase) is an enzyme that catalyzes the transfer of the gamma-phosphate from ATP to adenosine, and immune systems and inhibitors of the enzyme could play an important pharmacological role in increasing intravascular adenosine concentrations and acting as anti-inflammatory agents. ADA (Adenosine deaminase) is a protein-coding gene that encodes an enzyme that catalyzes the hydrolysis of adenosine to inosine in the purine catabolic pathway, and deaminase is an enzyme that detach amino group (-NH2) from nitrogen-containing bases (purines and pyrimidines) and their derivatives. They are often named after their substrate. Two well-known members of this group are adenosine deaminase and cytidine deaminase; PNP (Purine Nucleo side Phosphorylase). This gene encodes an enzyme that reversibly catalyzes the phosphorolysis of purine nucleosides. Mutations that result in nucleoside phosphorylase deficiency result in defective T-cell (cell-mediated) immunity but can also affect B-cell immunity and antibody responses. ADORA2A (Adenosine A2a Receptor), an adenosine receptor of the A2A subtype, uses adenosine as the preferred endogenous agonist and preferentially interacts with the G(s) and G(olf) family of G proteins to increase intracellular cAMP levels. It plays an important role in many biological functions, such as immune function and pain regulation. It has been implicated in pathophysiological conditions such as inflammatory diseases and neurodegenerative disorders. Furthermore, Figure 17E shows that KEGG analysis based on the DAVID database screened out a total of four signaling pathways, which were sequenced according to -Log P and the enriched genes of each pathway. The main pathways of α-arbutin enrichment include Nitrogen metabolism, Nucleotide metabolism, and Purine metabolism.

## 3. Discussion

The effect of α-arbutin on skin pigmentation has long been verified (Saeedi M et al., 2021) [32], but the protective effect and mechanism of α-arbutin on skin induced by UVB are not comprehensive. In addition, (Wang, Y et al., 2020) [33] showed that the arbutin in Rhodiola crenulatahas has antioxidant and anti-photoaging effects, but this study has some shortcomings. On the one hand, according to the structural formula given by the study, it is found that the object of their study is β-arbutin, which is different from α-arbutin in this study. Secondly, they only demonstrated that β-arbutin could effectively improve the apoptosis induced by UVB irradiation and regulate the production of inflammatory cytokines IL-6 and TNF- α, but there was no experimental study on arbutin in vivo. In this study, α-arbutin was utilized to investigate its anti aging effect on a mouse model of photoaging. The results of animal experiments showed that α-arbutin can significantly reduce the symptoms of erythema and wrinkles on the back of mouse skin caused by ultraviolet light. The results of HE staining and Masson staining showed that α-arbutin effectively improved UV-induced epidermal thickening and reduced dermal collagen fiber breakage. However, the results of immunohistochemistry and TB staining showed that α-arbutin could significantly reduce the aggregation of mast cells and down-regulate the expression of TNF-α, IL-6, and IL-1β, mitigating the occurrence of cellular inflammation. Collagen is the main frame structure of the extracellular matrix. Human skin collagen is mainly composed of type I collagen, and fibroblasts are the main cells that synthesize collagen. Type I collagen is the most common collagen, accounting for more than 70% of normal skin collagen. Collagen plays an important role in the normal physiological processes of the skin and the occurrence of diseases, such as wound healing and skin aging. In recent years, promoting collagen synthesis in fibroblasts for medical and cosmetic purposes has become a research hotspot. In this study, the immunohistochemistry analysis of mouse back skin tissue not only reduced the expression of inflammatory factors but also increased the expression level of COL-1, thereby supplementing the skin’s collagen content. Through network pharmacology studies, it has been found that α-arbutin and the cross-targets of photoaging and whitening are related to signaling pathways such as TYR. Through transcriptomic and metabolomic analysis, it is suggested that α-arbutin may regulate a variety of signaling pathways through multiple target proteins such as COL-1 and IL-6, IL-1β, IL-17, AMPK, and TNF-α, thereby affecting the anti aging response of the body. α-Arbutin can inhibit the activity of human tyrosinase and reduce the production of melanin. However, the high hydrophilicity and hygroscopic properties of alpha-arbutin result in inadequate absorption from the skin layer, thus reducing the therapeutic effectiveness of alpha-arbutin topical products (Zhu X et al., 2018) [23]. The results only discussed the therapeutic effect of α-arbutin on UVB-induced skin photoaging at a certain safe dose, and the specific action process of α-arbutin after entering the human body needs further study.

## 4. Materials and Methods

### 4.1. MTT Cytotoxic Assay

Hakata cells were used in the experiment, and the cells were extracted from liquid nitrogen for resuscitation. When the cells were in good viability, they were inoculated in 96-well plates at a density of 50,000 cells/mL, and 100 μL of cell suspension was added to each well. After 24 h, the cells adhered to the wall, discarded the former medium, and were supplemented with 100 μL of complete medium amended with 2 μg/mL, 1 μg/mL, and 0.5 μg/mL of α-arbutin, respectively. These cultures were incubated for another 24 h at a constant 37 °C and 5% CO_2_ in an environment-controlled incubator. Following this, 100 μL of MTT (0.5 mg/mL, base medium dilution) was added to a 96-well plate. With the aid of tin foil, it was placed in a culture box for 4 h before discarding the initial solution and adding 100 μL of DMSO to each well, which was then shaken for 10 min. The absorbance reading was acquired at 570 nm, calculating the survival rate of the cells.

### 4.2. Animals and Treatment

SPF-grade KM (Kunming) mice (5 weeks old, male, 20–25 g) were acquired from Guangdong Experimental Animal Center. Ethical approval for all experiments was granted by the Animal Ethics Committee, the Guangdong University of Technology. The α-arbutin (C_12_H_16_O_7_, purity ≥ 98%) used in the experiment was purchased from Aladdin, Shanghai. Mice were maintained under standard conditions (24 ± 1 °C temperature, 70–75% humidity, 12 h light/dark cycle) with free access to food and water. The day before the experiment, after 4 × 4 cm of hair removal on the backs of the mice. Fifty mice were randomly divided into 5 groups: control group, model group, 0.5% α-arbutin (model + AR − L), 1% α-arbutin (model + AR-M), and 2% α-arbutin (model + AR − H).

Among them, the mice in the control group were fed normally without any treatment, while the mice in the model group were irradiated with a UVB lamp for a certain period of time and smeared with a certain dose of normal saline (irradiation intensity 300 mj/days, irradiation every other day for 28 days, and saline 200 μL/day for 28 days). The mice in the AR − L group were irradiated with a certain dose of UVB lamp and smeared with a certain dose of drugs after irradiation (radiation intensity: 300 mj/days, every other day for 28 days; saline containing 0.5% α-arbutin 200 μL/each for 28 days), and mice in the AR m group were irradiated with a certain dose of UVB lamp and smeared with a certain dose of drugs after irradiation (radiation intensity 300 mj/day, every other day for 28 days; saline containing 1% α-arbutin 200 μL/each for 28 days). The mice in the AR − H group were irradiated with a certain dose of UVB lamp and smeared with a certain dose of drugs after irradiation (radiation intensity 300 mj/days, irradiation every other day for 28 days; smearing 200 μL of saline containing 2% α-arbutin per mouse/day for 28 days). On the 28th day after administration, the back skin of the mice was photographed and scored according to the visual scoring standard. Subsequently, the mice were euthanized by cervical dislocation, and the skin tissues from their backs were fixed and frozen using 4% paraformaldehyde.

### 4.3. Hematoxylin and Eosin Staining (H&E)

The skin tissue to be fixed was made into sections of paraffin and stained with hematoxylin and eosin. It was then examined under a microscope, and images were collected for histological analysis.

### 4.4. Toluidine Blue Staining

Paraffin sections underwent water dewaxing. Tissue sections were immersed in a dye solution for 2–5 min, followed by H_2_O washes, differentiation with 0.1% glacial acetic acid, drying, and transparent sealing. Observation under microscopy, image capture, and analysis.

### 4.5. Masson Staining

After paraffin sectioning of the tissue, soak in xylene and dehydrate with a gradient of anhydrous ethanol. They were then stained with Weigert-iron-hematoxylin for 10 min, followed by rinsing with distilled water. One percent hydrochloric acid alcohol was used for differentiation and then the tissue washed with distilled water. Stain with Ponceau S Staining Solution for 5–10 min. Treatment with 1% Phosphomolybdic acid for 1 min, then re-dye with Water blue for 5 min without water washing. Using 1% acetic acid treatment for 1 min and dehydrating with 95% anhydrous ethanol multiple times. Finally, they were transparentized with xylene and sealed with a neutral adhesive. Following the staining, collagen fibers will appear blue, cytoplasm and red blood cells will appear red, and the nucleus will appear blue-brown.

### 4.6. Immunohistochemistry

Paraffin sections at a concentration of 4 μM were dewaxed with xylene, gradually dehydrated with anhydrous ethanol, and then treated with 3% hydrogen peroxide for 10 min. Afterwards, they were washed with a phosphate-buffered solution. Following serum blockade, antibodies for COL-1, TNF-α, IL-6, and IL-1β (diluted with distilled water in a 1:100 volume ratio) were incubated overnight at 4 °C. The second antibody, a rabbit IgG enzyme-linked antibody, was then added, and the samples were incubated at 37 °C. After incubation with Horseradish peroxidase, DAB staining and Haematoxylin restaining were performed. After the production is completed, randomly select the shooting site under a 200× microscope. Apply the software Image-Pro Plus to measure the IOD value of COL-1, TNF-α, IL-6, and IL-1β. Finally, data were collected and plotted with GraphPad Prism (8.0.2).

### 4.7. Metabolomics Study

The chosen experimental approach for metabolomics was informed by earlier studies, albeit with minor alterations. To prepare the sample, the homogenate was prepared by mixing 0.1 g of skin tissue with 1 mL of chromatographic-grade methanol and spun at 13,000 g for 15 min at 4 °C. Subsequently, the supernatant underwent filtration with a 0.22 μm filter (NEST Biotechnology Wuxi Nest Life Technology Co., Ltd., Wuxi, China), followed by the addition of 100 μL of this supernatant to the vial for further analysis. Using an ultrahigh-performance liquid chromatography system to perform chromatographic separation of the target compound through a liquid chromatography column. Chromatography settings comprise the Agilent 1290 Infinity LC ultrahigh-performance liquid chromatography system (UPLC), a hydrophilic interaction liquid chromatographic (HILIC) column for separation at 25 °C, a flow rate of 0.5 mL/min and a 2 µL injection volume. The composition of the mobile phase ought to include A: water mixed with 25 mmol/L ammonium acetate and 25 mmol/L ammonia water, and B: acetonitrile. Gradual elution conditions should be maintained as follows: 0 → 0.5 min, 95% B; 0.5 min → 7 min, 95% → 65% B; 7 min → 8 min, 65% → 40% B; 8 min → 9 min, 40% B; 9 min → 9.1 min, 40% → 95% B; and 9.1 min → 12 min, 95% B. The AB Triple TOF 6600 mass spectrometer was employed for gathering both primary and secondary spectra of the specimen. Conditions for ESI source: Gas from Ion Source at a ratio of 1:60, Gas from Ion Source at 2:60, Current gas at 30, Voltage of IonSapary Floating: ±5500 V for both positive and negative modes; Mass Spectrometry (TOF) scan in mass-to-charge (*m*/*z*) range: 60–1000 Da, and ion scan of the product in mass-to-charge (*m*/*z*) range: 25–1000 Da; Secondary mass spectrometry was conducted using data-dependent acquisition and a high-sensitivity mode, featuring a clustering potential of ±60 V for both positive and negative modes, and a Collision Energy of (35 ± 15) eV.

### 4.8. Transcriptome Sequencing and Analysis

Transcriptome sequencing of library data was performed using the Illumina Hiseq 4000 platform. In order to ensure data quality, fast quality control is used to filter out low-quality data for offline data raw reads and remove sequences containing connectors, sequences containing more than 10% N (uncertain base), sequences containing base A, and sequences containing more than 50% of low-quality base content (Q ≤ 20). The Bowtie2 short sequence alignment tool was used for ribosome alignment, while HISAT2 v2. software (http://ccb.jhu.edu/software/hisat2/index.shtml accessed on 11 April 2024) was used for sequencing sequence alignment and reference genome alignment. Stringtie software (accessed on 11 April 2024) was used for transcript reconstruction, and the expression amount of all genes in each sample was calculated. Calculate the correlation of samples using the R language. Using DESeq software 1.26.0 (https://www.rdocumentation.org/packages/DEGseq/versions/1.26.0 (accessed on 11 April 2024)) to analyze differentially expressed genes, FDR < 0 Genes with 0.05 and |log2(FC)| > 1 were labeled as significantly different genes. Map differentially expressed proteins to various terms in the GO database, perform comparative tests, and perform GO enrichment analysis on differentially expressed genes. Compare sequencing results with the KEGG database to analyze their pathways and perform functional annotation and classification of differentially expressed genes.

### 4.9. Network Pharmacology Analysis

The search for the “senescence” keyword in the GeneCards database (www.genecards.org/ (accessed on 11 April 2024)) generated targets related to senescence. Potential targets of differential metabolites (DMs) were sourced from the SEA (https://sea.bkslab.org/ (accessed on 11 April 2024)) and TCMSP databases (http://tcmspw.com/tcmsp.php (accessed on 11 April 2024)) and their intersections with senescence were determined through Venny (https://bioinfogp.cnb.csic.es/tools/venny/index.html (accessed on 11 April 2024)). The tool STRING (www.string-db.org/ (accessed on 11 April 2024)) was used to examine both the direct and indirect interplays among these targets. Subsequently, a PPI network map was created, visualizing the top 20 targets of the highest degree. DAVID (https://david.ncifcrf.gov (accessed on 11 April 2024)) served the purpose of categorizing Gene Ontology (GO) and enriching pathways in the Kyoto Encyclopedia of Genes and Genomes (KEGG). Visualization of GO terms and KEGG pathways was achieved through the online tool Weishengxin (http://www.bio-informatics.com.cn/ (accessed on 11 April 2024)).

## 5. Conclusions

In summary, the study investigated the anti-photoaging and whitening effects of α-arbutin in animal photoaging models. Through animal experiments, network pharmacology, metabolomics, and transcriptomic analysis, it was found that α-arbutin has strong anti-photoaging and whitening abilities. This study found that α-arbutin can reduce the fracture of dermal collagen fibers, reduce the direct damage of ultraviolet radiation on dermal collagen, and reduce the expression of inflammatory factors in cells. The significant improvement in UV-induced cellular inflammation and the modulation of key metabolic pathways contribute to elucidating the mechanisms underlying the anti-photoaging and whitening effects of α-arbutin. 

## Figures and Tables

**Table 1:** Evaluation criteria of erythema and wrinkles on the back of mice.

Score	Characterization (Wrinkles)	Score	Characterization (Erythema)
1	Smooth skin without wrinkles	1	Smooth skin
2	There are fine marks	2	A few erythema or scabs
3	A small amount of shallow wrinkles	3	Moderate erythema or scab
4	A large number of superficial wrinkles	4	Erythema and scab seriously distributed
5	Thickened skin and deep wrinkles	5	Skin erosion

## Data Availability

The original contributions presented in the study are included in the article, further inquiries can be directed to the corresponding authors.
